# Personalized screening based on risk and density: prevalence data from the RIBBS study

**DOI:** 10.1007/s11547-025-01981-5

**Published:** 2025-03-21

**Authors:** Francesca Caumo, Gisella Gennaro, Alessandra Ravaioli, Enrica Baldan, Elisabetta Bezzon, Silvia Bottin, Paolo Carlevaris, Lina Ciampani, Alessandro Coran, Chiara Dal Bosco, Sara Del Genio, Alessia Dalla Pietà, Fabio Falcini, Federico Maggetto, Giuseppe Manco, Tiziana Masiero, Maria Petrioli, Ilaria Polico, Tiziana Pisapia, Martina Zemella, Manuel Zorzi, Stefania Zovato, Lauro Bucchi

**Affiliations:** 1 Breast Radiology Unit, Department of Imaging and Radiotherapy, Veneto Institute of Oncology (IOV) IRCCS, Via Gattamelata 64, 35128 Padua, Italy; 2 Emilia‑Romagna Cancer Registry, Romagna Cancer Institute IRCCS Istituto Romagnolo per lo Studio dei Tumori (IRST) Dino Amadori, Meldola, Forlì, Italy; 3 Cancer Prevention Unit, Local Health Authority, Forlì, Italy; 4 AORN A. Cardarelli, Naples, Italy; 5 SER - Servizio Epidemiologico Regionale e Registri Azienda Zero, Padua, Italy; 6 Hereditary Tumors Unit, Veneto Institute of Oncology (IOV) IRCCS, Padua, Italy

**Keywords:** Breast cancer, Cancer screening, Breast density, Risk assessment, Digital breast tomosynthesis

## Abstract

**Purpose:**

To present the prevalence screening results of the RIsk-Based Breast Screening (RIBBS) study (ClinicalTrials.gov NCT05675085), a quasi-experimental population-based study evaluating a personalized screening model for women aged 45–49. This model uses digital breast tomosynthesis (DBT) and stratifies participants by risk and breast density, incorporating tailored screening intervals with or without supplemental imaging (ultrasound, US, and breast MRI), with the goal of reducing advanced breast cancer (BC) incidence compared to annual digital mammography (DM).

**Materials and methods:**

An interventional cohort of 10,269 women aged 45 was enrolled (January 2020–December 2021. Participants underwent DBT and completed a BC risk questionnaire. Volumetric breast density and lifetime risk were used to assign five subgroups to tailored screening regimens: low-risk low-density (LR–LD), low-risk high-density (LR–HD), intermediate-risk low-density (IR–LD), intermediate-risk high-density (IR–HD), and high-risk (HR). Screening performance was compared with an observational control cohort of 43,838 women undergoing annual DM.

**Results:**

Compared to LR–LD, intermediate-risk groups showed a 4.9- (IR–LD) and 4.6-fold (IR–HD) higher prevalence of BC, driven by a 7.1- and 7.1-fold higher prevalence of pT1c tumors. The interventional cohort had lower recall rate (rate ratio, 0.5), higher surgery rate (1.9) and increased prevalence of DCIS (2.9), pT1c (2.3) and grade 3 tumors (2.4), compared to controls.

**Conclusion:**

The prevalence screening demonstrated the feasibility of using DBT and —in high-density subgroups— supplemental US. The stratification criteria effectively identified subpopulations with different BC prevalence. Increasing the detection rate of pT1c tumors is not sufficient but necessary to achieve a reduction in advanced BC incidence.

## Introduction

In the risk-stratified breast cancer (BC) screening strategy [1–4], personal risk estimates for developing the disease within a specific time frame inform the choices regarding screening intervals, starting age, and imaging modalities [5]. This approach aligns with the broader trend towards precision medicine, paralleling the shift towards personalized treatment for BC [6]. Risk stratification is increasingly recognized as a crucial factor in optimizing the balance between the benefits and harms of BC screening, especially in younger women [7].

The latest European Guidelines (ECIBC) provided a conditional recommendation for mammography screening for women aged 45–49 years, with no definitive guidelines established regarding screening intervals. Both biennial and triennial screening are preferred over annual screenings, with the optimal screening interval still regarded as an area for research [8]. In this context, effective stratification may help to target the appropriate individuals for screening, thereby reducing breast cancer mortality, preventing more aggressive treatments, and improving overall quality of life. At the same time, it can mitigate the risks associated with screening, such as radiation exposure, frequent recalls, unnecessary surgical referrals, biopsies, false-positive and false-negative results, and overdiagnosis [9–11].

In the last decades, several risk prediction models have been developed, integrating many BC risk factors [5, 12, 13]. Mammographic breast density has been increasingly incorporated into these models to improve their predictive power [14–16]. Breast density not only obscures tumors, thereby increasing interval cancer rates [17, 18], but also serves as an independent risk factor beyond masking effects [19–21]. This has led to the recommendations for supplemental imaging in women with dense breasts [22–24].

For large-scale implementation, personalization of screening protocols requires a thorough evaluation of effectiveness. There are unanswered questions regarding how well different stratified protocols compare with traditional screening approaches [25–27], as well as how these protocols perform in real-world settings [28, 29]. While randomized controlled trials have addressed the first question [4, 30], observational studies may provide essential insights into their effectiveness in the public health context.

To address the critical need to improve BC screening, we designed the Risk-Based Breast Screening (RIBBS) study. This quasi-experimental, population-based study targets women aged 45–49 years and aims to evaluate the potential benefits of a screening model that stratifies participants based on personal risk and breast density. This model integrates digital breast tomosynthesis (DBT) with ultrasound (US) and breast MRI when required by breast density and/or risk, at tailored intervals. DBT allows better visualization of overlapping tissue [31], US acts as an effective adjunct for detecting cancers in dense breasts [32], and MRI is used for high-risk populations due to its high sensitivity [33]. The study compares this model with standard annual screening using digital mammography (DM) to test whether the personalized screening model reduces the cumulative incidence of advanced BC.

In this paper, we present the results of the initial prevalence screening, with the objectives of (1) assessing the feasibility of implementing the RIBBS screening model, (2) evaluating the effectiveness of stratification criteria in identifying subpopulations with different prevalence of BC detectable by screening, and (3) determining whether the initial performance measures suggest a potential for reducing the incidence of advanced BC in subsequent screening rounds.

## Materials and methods

This quasi-experimental study, approved by the Institutional Ethics Committee of the Veneto Institute of Oncology (IOV) IRCCS, Padua, Italy (approval code: “RIBBS 2019/37”), is registered at ClinicalTrials.gov (registration no. NCT05675085). The design compares a prospective interventional cohort with a non-equivalent observational control cohort [34]. All women in the prospective interventional cohort provided written informed consent. In contrast, informed consent was not required for the women in the retrospective observational cohort because their participation in the population-based screening program was separate from this study, and the ethics committee felt that additional consent was not necessary for this observational analysis.

### Study design and participants

#### Prospective interventional cohort

Only a limited number of Italian Regions have implemented breast cancer screening programs specifically targeting women aged 45–49. The Veneto Region in northern Italy is not among these regions. In 2018, we proposed the RIBBS study [35] to the Veneto Region Department of Health, which approved it as a pilot project in two provinces, Padua and Rovigo. Due to the relatively small size of the population annually available for the study (approximately 10,000 women), random assignment to multiple screening arms was not statistically feasible. In addition, this approach was considered unacceptable by public health authorities in a prevention context.

To address these constraints, a quasi-experimental design was adopted, facilitated by collaboration with screening authorities in three provinces of the neighboring Emilia-Romagna Region, where women aged 45–49 years have been regularly invited to annual digital mammography (DM) since 2010 [36].

Women aged 45 years residing in the provinces of Padua and Rovigo (Veneto region, Italy), without a history of breast cancer or known germline mutations in BRCA1/2, PALB2, TP53 or equivalent genes were invited to participate in a ‘personalized screening program’ at the IOV (Padua, Italy). Women were enrolled from January 2020 to December 2021. During prevalence screening, participants underwent bilateral two-view DBT and completed a risk questionnaire. DBT images were processed with automated software (Volpara v. 1.5.5.1) to calculate volumetric breast density (VBD) [37, 38].

A breast density threshold of VBD = 25% was identified as a practical cut-off to balance the benefits of additional imaging with resource utilization and clinical feasibility. This threshold was determined using a preliminary set of mammograms from women aged 45–49 years in which VBD was measured with the same software used in the RIBBS study.  In this preliminary phase, an experienced breast radiologist also assessed the images and the need for additional ultrasound based on the masking effect of dense tissue. However, during the RIBBS study itself, the 25% VBD threshold was applied directly based on quantitative measurements, without radiologist-based evaluation of masking.

Women with a mean VBD greater than 25% were invited for supplemental US. Individual risk factors and mean VBD were used to estimate personal lifetime risk (LTR) using the Tyrer-Cuzick risk prediction model [12, 39]. Based on breast density and personal LTR, participants were categorized into five subgroups:

1. Low-risk, low-density (LR–LD): LTR ≤ 17% and mean VBD < 25%, recommended for biennial screening with DBT (‘2yDBT’ protocol);
2. Low-risk, high-density (LR–HD): LTR ≤ 17% and mean VBD ≥ 25%, recommended for biennial screening with DBT and US [‘2y(DBT + US)’ protocol];
3. Intermediate-risk, low-density, (IR–LD): LTR between 17 and 30%, or LTR > 30% without family history, and mean VBD < 25%, recommended for annual DBT screening (‘1yDBT’ protocol);
4. Intermediate-risk, high-density (IR–HD): women with LTR between 17 and 30%, or LTR > 30% without family history, and mean VBD ≥ 25%, recommended for annual screening with DBT and US [‘1y(DBT + US)’ protocol];
5. High-risk (HR): women with LTR > 30% and a family history of breast cancer, recommended for annual DBT and MRI, regardless of breast density.

All initial DBT studies were interpreted independently by two breast radiologists. In case of imaging abnormality, women were recalled for further investigation and, if necessary, biopsy. Confirmed BC cases followed post-treatment plans.

In subsequent rounds of screening, still ongoing, subgroups 1 and 3 continue with double readings of DBT studies, while subgroups 2, 4, and 5 receive single radiologist reading for either (DBT + US) or (DBT + MRI) studies. In fact, at the time of recruitment, breast density and individual risk were not yet known, as they were assessed only after the first DBT and the information collected from the Tyrer-Cuzick questionnaire. Consequently, during the first screening round, all DBT examinations were double read, and women with VBD ≥ 25% returned for supplemental US. However, in subsequent screening rounds, each woman was already assigned to her respective density/risk subgroup. Therefore, double reading was maintained for those following protocols based on DBT alone, while for those in protocols including DBT+US or DBT+MRI, a single reader interpreted the DBT. This approach was adopted because the additional imaging modalities (US or MRI) provided complementary diagnostic information, mitigating the risk of misinterpretation that justifies double reading when DBT is the only imaging modality used. The detailed protocol of the RIBBS study has been published previously [35].

#### Retrospective observational cohort

Emilia-Romagna is one of the few regions in Italy that has implemented a population-based screening program for women aged 45–49, using annual DM in two-views with double reading. The observational retrospective cohort included only women aged 45 years participating in the Emilia-Romagna screening program (provinces of Ravenna, Forlì-Cesena and Rimini) who received their prevalence screen between January 2013 and December 2020. The inclusion/exclusion criteria were identical to those of the prospective cohort. However, breast density and risk estimation were not collected, as these parameters were not part of the ‘one-size-fits-all’ screening program [40].

### Statistical analysis

A complete set of standard screening outcomes was calculated, including rates per thousand and percentage proportions. These outcomes included recall rate, stratified into noninvasive and invasive assessment, rates of women referred and undergoing surgery, cancer detection rates, further stratified into ductal carcinoma in situ (DCIS) and invasive cancer rates, and positive predictive values.

For each outcome, we calculated the ratio and 95% confidence interval (CI) between each rate/percentage value for LR–HD, IR–LD, IR–HD, HR subgroups of the prospective interventional cohort and the corresponding rate/percentage of the reference subgroup of LR–LD women undergoing 2-year DBT. This analysis aimed to evaluate the effectiveness of the RIBBS model in identifying women at different risks for breast cancer.

Similarly, ratios and 95%CI between each rate and percentage value in the overall prospective interventional cohort and the corresponding one in the observational control cohort were calculated to analyze the differences between the two screening models.

*P* values were calculated using chi-square tests for heterogeneity with 1 degree of freedom, with a significance level set at *P* < 0.05.

The comparison of invasive BC detection rates between the two cohorts was not adjusted for differences in incidence observed in the 5 years prior to recruitment [35]. This decision was based on the fact that the incidence rate ratio between the interventional cohort and the observational control cohort was slightly less than one (0.96; 95% CI 0.83–1.11) and not statistically significant.

The statistical analysis was performed using Stata Statistical Software Release 15 (StataCorp LP, College Station, TX), and the plots were generated with OriginPro 2020b (OriginLab Corporation, Northampton, MA).

## Results

### Study population

**Fig. 1:**
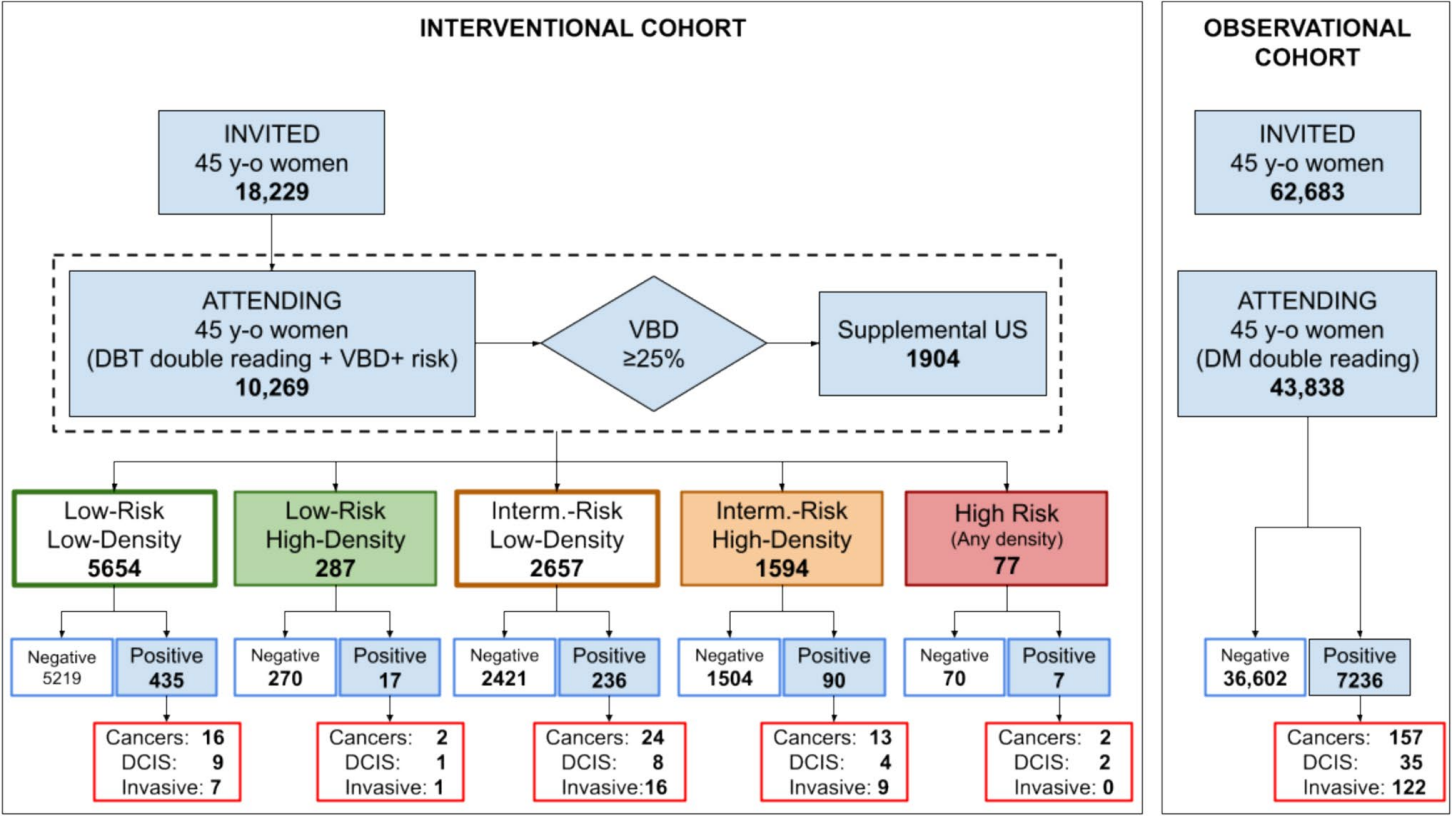
Prevalence screen flowchart of the RIBBS study. *Note* DBT, digital breast tomosynthesis; DM, digital mammography; VBD, volumetric breast density; DCIS, ductal carcinoma in situ

Of the 18,229 women aged 45 years invited, 10,269 (56.3%) agreed to participate in the interventional cohort of the RIBBS study. The observational cohort included 43,838 women aged 45 years, representing 69.9% of those invited. Overall, the study population included 54,107 women. Figure 1 illustrates the prevalence screen flowchart and provides the outcomes of the baseline examinations (DBT for the interventional cohort and DM for the observational cohort).

Table 1 presents the stratification by breast density into two categories and breast cancer risk into three categories for women participating in the interventional cohort. Age is not reported because all participants were 45 years old.

**Table 1:** Stratification by breast density and breast cancer risk of women participating in the interventional cohort

Characteristic	Number	Percentage (%)
Recruited	10,269	100
Breast density		
Low	8365	81.5
High	1904	18.5
Risk		
Low	5941	57.9
Intermediate	4251	41.4
High	77	0.7

Most of the recruited women had low-density breasts (81.5%, 8365/10,269) and were at low risk for breast cancer (57.9%, 5941/10,269). Women at intermediate risk were 41.4% (4251/10,269), while only 0.7% (77/10,269) were classified as high risk. Overall, 18.5% of women had dense breasts and underwent supplemental US.

**Fig. 2:**
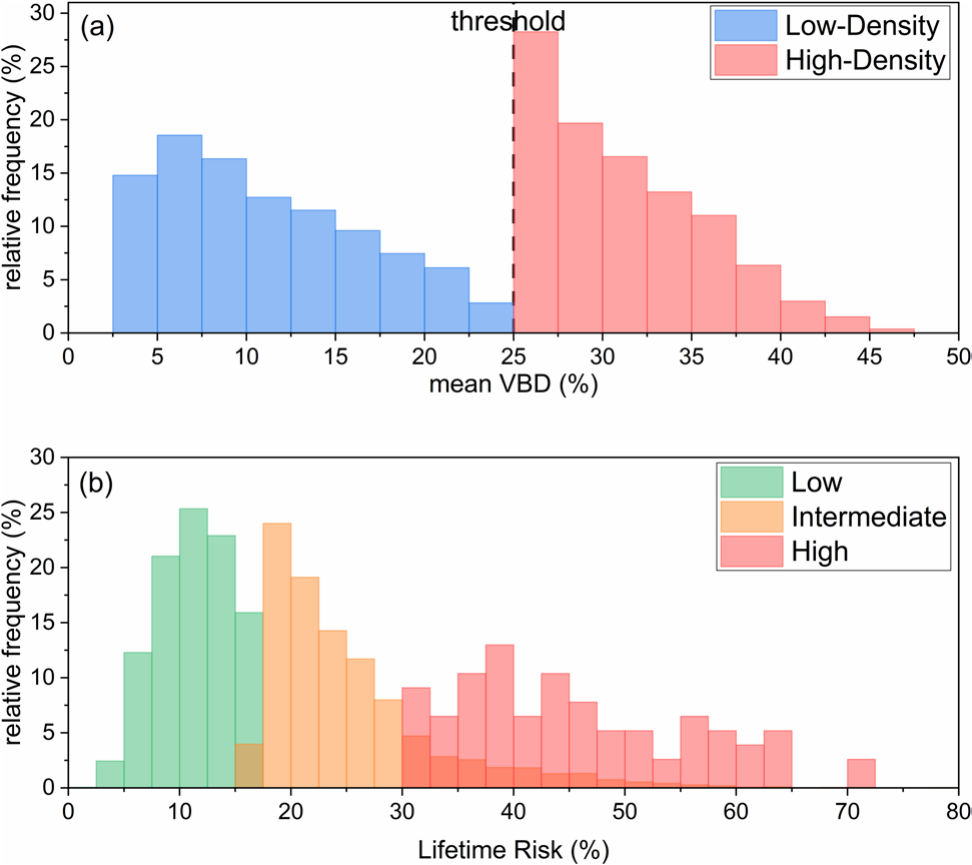
**a** Normalized histogram of mean VBD (averaged across four views) for all women recruited in the interventional cohort of the RIBBS study. The dashed vertical line represents the VBD threshold to distinguish women in the high-density subgroups receiving supplemental US from those in the low-density subgroup; **b** normalized histogram of personal LTR estimated using the Tyrer-Cuzik risk model for all women recruited in the interventional cohort of the RIBBS study. *Note*. VBD, volumetric breast density; RIBBS, risk-based breast screening; US, ultrasound; LTR, lifetime risk

Figure 2 presents the normalized histograms of mean volumetric breast density for the low- and high-density subgroups (a) and personal lifetime risk for the three risk subgroups (b) in all women recruited into the interventional cohort. The distributions are shown separately by breast density and risk category, respectively.

### Screening outcome measures

Table 2 presents screening outcomes as rates (n° of women per 1,000) and absolute numbers for both the interventional and observational cohorts. For the interventional cohort, results are reported overall and detailed for the five subgroups defining the different rescreening protocols. In addition, it includes the positive predictive values of recall (PPV1) and biopsy (PPV3).

**Table 2:** Rates of women (per 1000) recalled for assessment, who underwent assessment, were referred for surgery, underwent surgery, and were diagnosed with B2 and B3 lesions, ductal carcinoma in situ (DCIS), and invasive breast cancer. Positive predictive values of recall and biopsy are also shown in the five risk and breast density subgroups of the prospective interventional cohort and the observational control cohort of the RIBBS study. Absolute numbers are provided in parentheses

	Prospective interventional cohort						Retrospective observational cohort
	LR–LD	LR–HD	IR–LD	IR–HD	HR	Overall	
Screened women	5654	287	2657	1594	77	10,269	43,838
Screening outcome (n° of women/1000)							
Recalled for assessment	76.9 (435)	59.2 (17)	88.8 (236)	56.5 (90)	90.9 (7)	76.4 (785)	165.1 (7236)
Undergoing assessment	76.6 (433)	59.2 (17)	88.8 (236)	55.8 (89)	90.9 (7)	76.2 (782)	159.4 (6987)
Non-invasive	57.3 (324)	17.4 (5)	58.7 (156)	14.4(23)	26.0 (2)	49.7 (510)	134.1 (5878)
Invasive	19.3 (109)	41.8 (12)	30.1 (80)	41.4 (66)	64.9 (5)	26.5 (272)	25.3 (1109)
B2 lesions	12.2 (69)	24.4 (7)	16.9 (45)	28.2 (45)	39.0 (3)	16.5 (169)	8.7 (383)
B3 lesions	4.4 (25)	3.5 (1)	5.3 (14)	4.4 (7)	– (0)	4.6 (47)	1.4 (61)
Referred for surgery	5.3 (30)	7.0 (2)	13.5 (36)	10.0 (16)	26.0 (2)	8.4 (86)	5.2 (227)
Undergoing surgery	5.3 (30)	7.0 (2)	12.8 (34)	10.0 (16)	26.0 (2)	8.2 (84)	4.4 (193)
Total detected cancers	2.8 (16)	7.0 (2)	9.0 (24)	8.2 (13)	26.0 (2)	5.6 (57)	3.6 (157)
DCIS	1.6 (9)	3.5 (1)	3.0 (8)	2.5 (4)	26.0 (2)	2.3 (24)	0.8 (35)
Low-grade	0.2 (1)	– (0)	1.1 (3)	– (0)	26.0 (2)	0.6 (6)	0.2 (8)
Intermediate-grade	0.4 (2)	3.5 (1)	1.1 (3)	0.6 (1)	– (0)	0.7 (7)	0.2 (10)
High-grade	1.1 (6)	– (0)	0.8 (2)	1.9 (3)	– (0)	1.1 (11)	0.3 (13)
Invasive cancers	1.2 (7)	3.5 (1)	6.0 (16)	5.6 (9)	– (0)	3.2 (33)	2.8 (122)
pT1mic-a-b	0.7 (4)	– (0)	1.9 (5)	0.6 (1)	– (0)	1.0 (10)	1.2 (53)
pT1c	0.5 (3)	3.5 (1)	3.8 (10)	3.8 (6)	– (0)	1.9 (20)	0.8 (37)
pT2-4	– (0)	– (0)	0.4 (1)	1.3 (2)	– (0)	0.3 (3)	0.6 (27)
pN-negative	0.7 (4)	3.5 (1)	4.1 (11)	5.6 (9)	– (0)	2.4 (25)	1.8 (78)
pN-positive	0.5 (3)	– (0)	1.5 (4)	– (0)	– (0)	0.7 (7)	0.8 (37)
Grade 1	0.4 (2)	– (0)	1.1 (3)	1.3 (2)	– (0)	0.7 (7)	0.5 (20)
Grade 2	0.5 (3)	3.5 (1)	2.6 (7)	1.3 (2)	– (0)	1.3 (13)	1.7 (75)
Grade 3	0.4 (2)	– (0)	2.3 (6)	3.1 (5)	– (0)	1.3 (13)	0.5 (23)
Percent proportions							
PPV1	1.6	5.9	6.8	10.0	–	4.2	1.7
PPV3	23.3	50.0	44.4	56.3	–	38.4	53.7

*Note* LR–LD, low-risk low-density; LR–HD, low-risk high-density; IR–LD, intermediate-risk low-density; IR–HD, intermediate-risk high-density; HR, high-risk (any density); B2 lesions, benign lesions from percutaneous biopsy; B3 lesions, lesions of uncertain malignant potential from percutaneous biopsy; DCIS, ductal carcinoma in situ; PPV1, positive predictive value of recall for additional imaging (non-invasive assessment); PPV3, positive predictive value of biopsy

It can be seen that the subgroup of low-risk women with dense breasts was very small (2.8%, 287/10,269), as the inclusion of VBD ≥ 25% as a risk factor in the Tyrer-Cuzick risk model often pushed the LTR beyond the 17% threshold for intermediate risk. Another small subgroup consists of high-risk women (monitored with annual DBT + MRI), as was expected.

### The discriminative ability of the personalization criteria

Categorizing the women in the interventional cohort, based on risk and breast density, into five subgroups associated with specific screening protocols for subsequent rounds allowed us to assess the discriminative ability of the personalization criteria chosen in the RIBBS study. Table 3 presents the ratio and relative 95% CI for each screening outcome, between LR–HD, IR–LD, IR–HD, HR subgroups with the low-risk low-density subgroup, which serves as the reference and undergoes biennial DBT rescreening.

**Table 3:** Screening outcome ratios and 95% confidence intervals of LR–HD, IR–LD, IR–HD, HR subgroups in the prospective interventional cohort to the LR–LD subgroup as reference (ref)

Screening outcome ratio	Prospective interventional cohort				
	LR–LD	LR–HD	IR–LD	IR–HD	HR
Recalled for assessment	1.0 (ref)	0.8 (0.5, 1.2)	1.2 (1.0, 1.3)	0.7 (0.6, 0.9)*	1.2 (0.6, 2.4)
Undergoing assessment	1.0 (ref)	0.8 (0.5, 1.2)	1.2 (1.0, 1.4)	0.7 (0.6, 0.9)*	1.2 (0.6, 2.4)
Non-invasive	1.0 (ref)	0.3 (0.1, 0.7)*	1.0 (0.9, 1.2)	0.3 (0.2, 0.4)*	0.5 (0.1, 1.8)
Invasive	1.0 (ref)	2.2 (1.2, 3.9)*	1.6 (1.2, 2.1)*	2.2 (1.6, 2.9)*	3.4 (1.4, 8.0)*
B2 lesions	1.0 (ref)	2.0 (0.9, 4.3)	1.4 (1.0, 2.0)	2.3 (1.6, 3.4)*	3.2 (1.0, 9.9)*
B3 lesions	1.0 (ref)	0.8 (0.1, 5.8)	1.2 (0.6, 2.3)	1.0 (0.4, 2.3)	–
Referred for surgery	1.0 (ref)	1.3 (0.3, 5.5)	2.6 (1.6, 4.1)*	1.9 (1.0, 3.5)*	4.9 (1.2, 20.1)*
Undergoing surgery	1.0 (ref)	1.3 (0.3, 5.5)	2.4 (1.5, 3.9)*	1.9 (1.0, 3.5)*	4.9 (1.2, 20.1)*
Total detected cancers	1.0 (ref)	2.5 (0.6), 10.7)	3.2 (1.7, 6.0)*	2.9 (1.4, 6.0)*	9.2 (2.2. 39.2)*
DCIS	1.0 (ref)	2.2 (0.3, 17.2)	1.9 (0.7, 4.9)	1.6 (0.5, 5.1)	16.3 (3.6,74.3)*
Low-grade	1.0 (ref)	–	6.4 (0.7, 61.3)	–	146.9 (13.5, 1602.6)*
Intermediate-grade	1.0 (ref)	9.9 (0.9, 108.3)*	3.2 (0.5, 19.1)	1.8 (0.2, 19.6)	–
High-grade	1.0 (ref)	–	0.7 (0.1, 3.5)	1.8 (0.4, 7.1)	–
Invasive cancers	1.0 (ref)	2.8 (0.4, 22.8)	4.9 (2.0, 11.8)*	4.6 (1.7, 12.2)*	–
pT1mic-a-b	1.0 (ref)	–	2.7 (0.7, 9.9)	0.9 (0.1, 7.9)	–
pT1c	1.0 (ref)	6.6 (0.7, 62.9)	7.1 (2.0, 25.8)*	7.1 (1.8, 28.3)*	–
pT2-4	1.0 (ref)	–	–	–	–
pN-negative	1.0 (ref)	4.9 (0.6, 43.9)	5.9 (1.9, 18.4)*	8.0 (2.5, 25.9)*	–
pN-positive	1.0 (ref)	–	2.8 (0.6. 12.7)	–	–
Grade 1	1.0 (ref)	–	3.2 (0.5, 19.1)	3.6 (0.5, 25.2)	–
Grade 2	1.0 (ref)	6.6 (0.7, 62.9)	5.0 (1.3, 19.2)*	2.4 (0.4, 14.1))	–
Grade 3	1.0 (ref)	–	6.4 (1.3, 31.3)*	8.9 (1.7, 45.7)*	–
Percent proportions					
PPV1	1.0 (ref)	3.7 (0.5, 28.1)	4.2 (1.8, 10.1)*	6.2 (2.4, 16.3)*	–
PPV3	1.0 (ref)	2.1 (0.5, 9.9)	1.9 (0.9, 4.0)	2.4 (1.1, 5.3)*	–

*Note* LR–LD, low-risk low-density; LR–HD, low-risk high-density; IR–LD, intermediate-risk low-density; IR–HD, intermediate-risk high-density; HR, high-risk (any density); B2 lesions, benign lesions from percutaneous biopsy; B3 lesions, lesions of uncertain malignant potential from percutaneous biopsy; DCIS, ductal carcinoma in situ; PPV1, positive predictive value of recall for additional imaging (non-invasive assessment); PPV3, positive predictive value of biopsy.

*Indicate a *p* value < .05

**Fig. 3:**
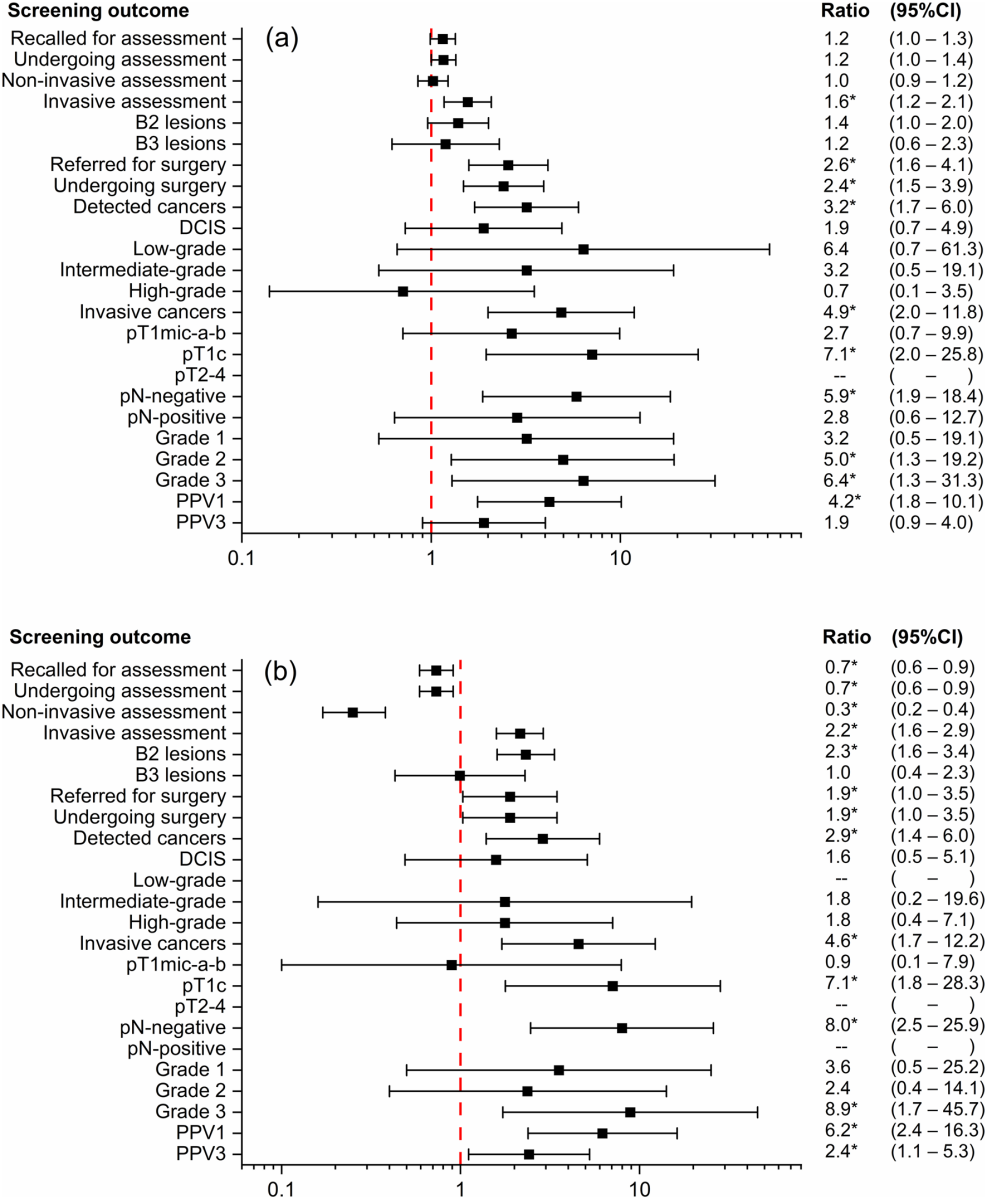
Comparison between screening outcomes for the two subgroups at intermediate risk **a** low-density, and **b** high-density, and the ‘low-risk low-density’ subgroup: forest plots of ratios between rates/percentages. *Note*. B2 lesions, benign lesions from percutaneous biopsy; B3 lesions, lesions of uncertain malignant potential from percutaneous biopsy; DCIS, ductal carcinoma in situ; PPV1, positive predictive value of recall for additional imaging (non-invasive assessment); PPV3, positive predictive value of biopsy

Because of the small numbers in the second (low-risk high-density) and fifth (high-risk) subgroups, comparison with forest plots depicted in Fig. 3 was limited to the two intermediate-risk subgroups. Each square symbol on the graph represents the ratio value for a given parameter, and the horizontal interval is the 95% CI of the ratio. The ratio values and CIs are also shown on the right side of the graph, with an asterisk when the difference between the two rates/percentages is statistically significant.

Women in the ‘intermediate-risk, high-density’ category were recalled less frequently for assessment, especially for noninvasive assessment. In contrast, women in both ‘intermediate-risk’ categories, as well as those in the high-risk category, had significantly higher rates of surgery, with rates doubled or higher. Compared with the reference group, the cancer detection rate was higher in the intermediate-risk subgroups, as were the rates of invasive cancers, pT1c and grade 3 cancers, confirming the role of risk assessment in screening stratification.

**Fig. 4:**
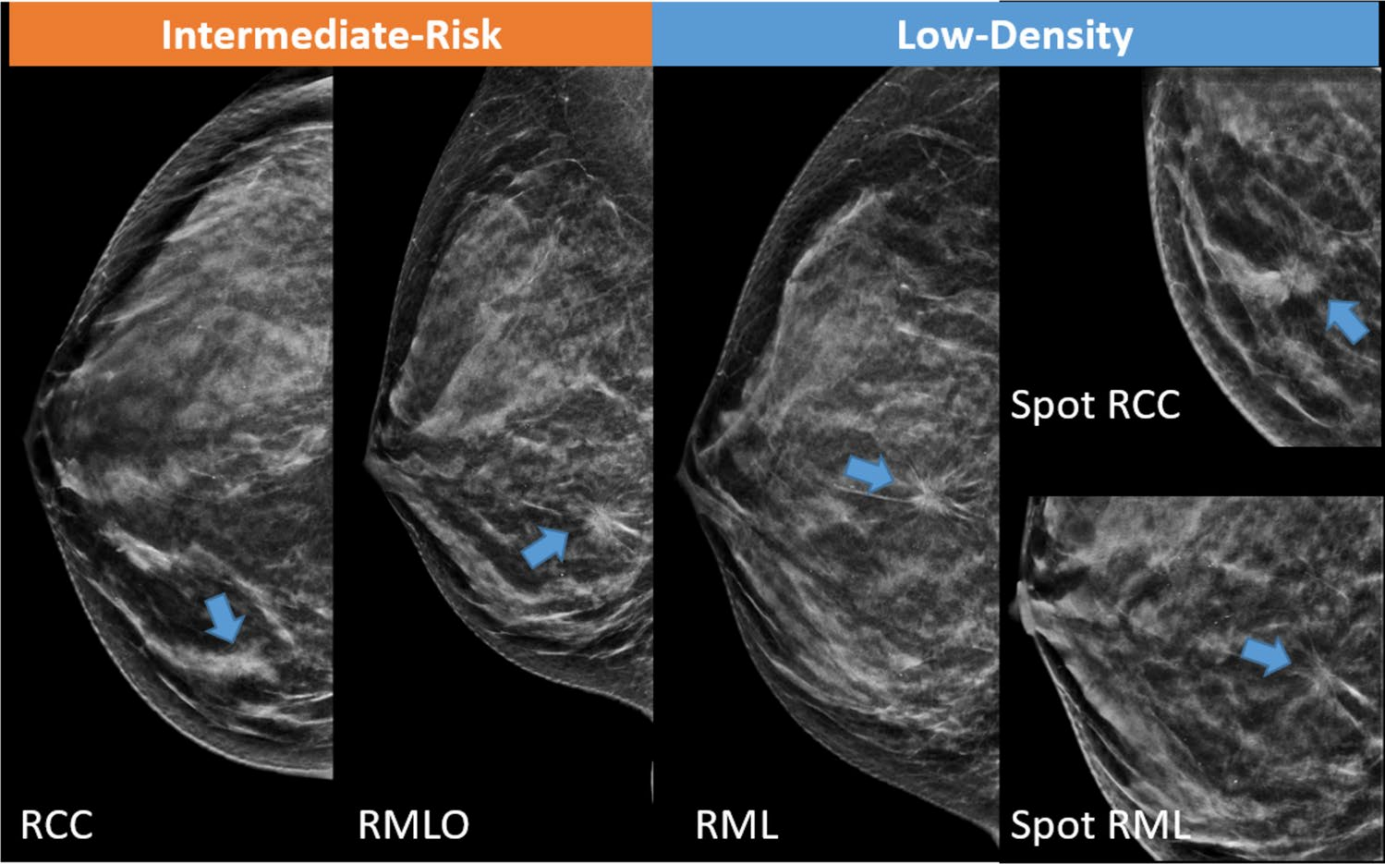
Example case of a grade 3 pT1c tumor in the right breast of an intermediate-risk woman with low-density breasts: RCC, RMLO, RML contact views, and RCC and RML spot views. Synthetic images obtained from DBT. The lesion was a spiculated mass, which on percutaneous biopsy proved to be a grade 3 IDC at the, confirmed by surgery. *Note*. RCC, right cranio-caudal; RMLO, right mediolateral-oblique; RML, right mediolateral; DBT, digital breast tomosynthesis; IDC, invasive ductal carcinoma

An example case of a grade 3 pT1c tumor in the right breast of an intermediate-risk woman with low-density breasts is shown in Fig. 4.

### Comparison between interventional and observational cohorts

Table 4 shows the ratio and 95%CI for each screening outcome between the entire prospective interventional cohort and the retrospective observational cohort taken as a reference.

**Table 4:** Screening outcome ratios and 95% confidence intervals of the prospective interventional cohort to the retrospective observational cohort as a reference (ref)

Screening outcome ratio	Prospective interventional cohort	Retrospective observational cohort
Recalled for assessment	0.5 (0.4, 0.5)*	1.0 (ref)
Undergoing assessment	0.5 (0.5, 0.51)*	1.0 (ref)
Non-invasive	0.4 (0.3, 0.4)*	1.0 (ref)
Invasive	1.1 (0.9, 1.2)	1.0 (ref)
B2 lesions	1.9 (1.6, 2.3)*	1.0 (ref)
B3 lesions	3.3 (2.3, 4.8)*	1.0 (ref)
Referred for surgery	1.6 (1.3, 2.1)*	1.0 (ref)
Undergoing surgery	1.9 (1.4, 2.4)*	1.0 (ref)
Total detected cancers	1.6 (1.2, 2.1)*	1.0 (ref)
DCIS	2.9 (1.7, 4.9)*	1.0 (ref)
Low-grade	3.2 (1.1, 9.2)*	1.0 (ref)
Intermediate-grade	3.0 (1.1, 7.9)*	1.0 (ref)
High-grade	3.6 (1.6, 8.1)*	1.0 (ref)
Invasive cancers	1.2 (0.8, 1.7)	1.0 (ref)
pT1mic-a-b	0.8 (0.4, 1.6)	1.0 (ref)
pT1c	2.3 (1.3, 4.0)*	1.0 (ref)
pT2-4	0.5 (0.1, 1.6)	1.0 (ref)
pN-negative	1.4 (0.9, 2.2)	1.0 (ref)
pN-positive	0.8 (0.4, 1.8)	1.0 (ref)
Grade 1	1.5 (0.6, 3.5)	1.0 (ref)
Grade 2	0.7 (0.4, 1.3)	1.0 (ref)
Grade 3	2.4 (1.2, 4.8)*	1.0 (ref)
Percent proportions		
PPV1	2.5 (1.7, 3.6)*	1.0 (ref)
PPV3	0.7 (0.5, 1.0)*	1.0 (ref)

*Note*. B2 lesions, benign lesions from percutaneous biopsy; B3 lesions, lesions of uncertain malignant potential from percutaneous biopsy; DCIS, ductal carcinoma in situ; PPV1, positive predictive value of recall for additional imaging (non-invasive assessment); PPV3, positive predictive value of biopsy

*Indicate a *p* value < .05

**Fig. 5:**
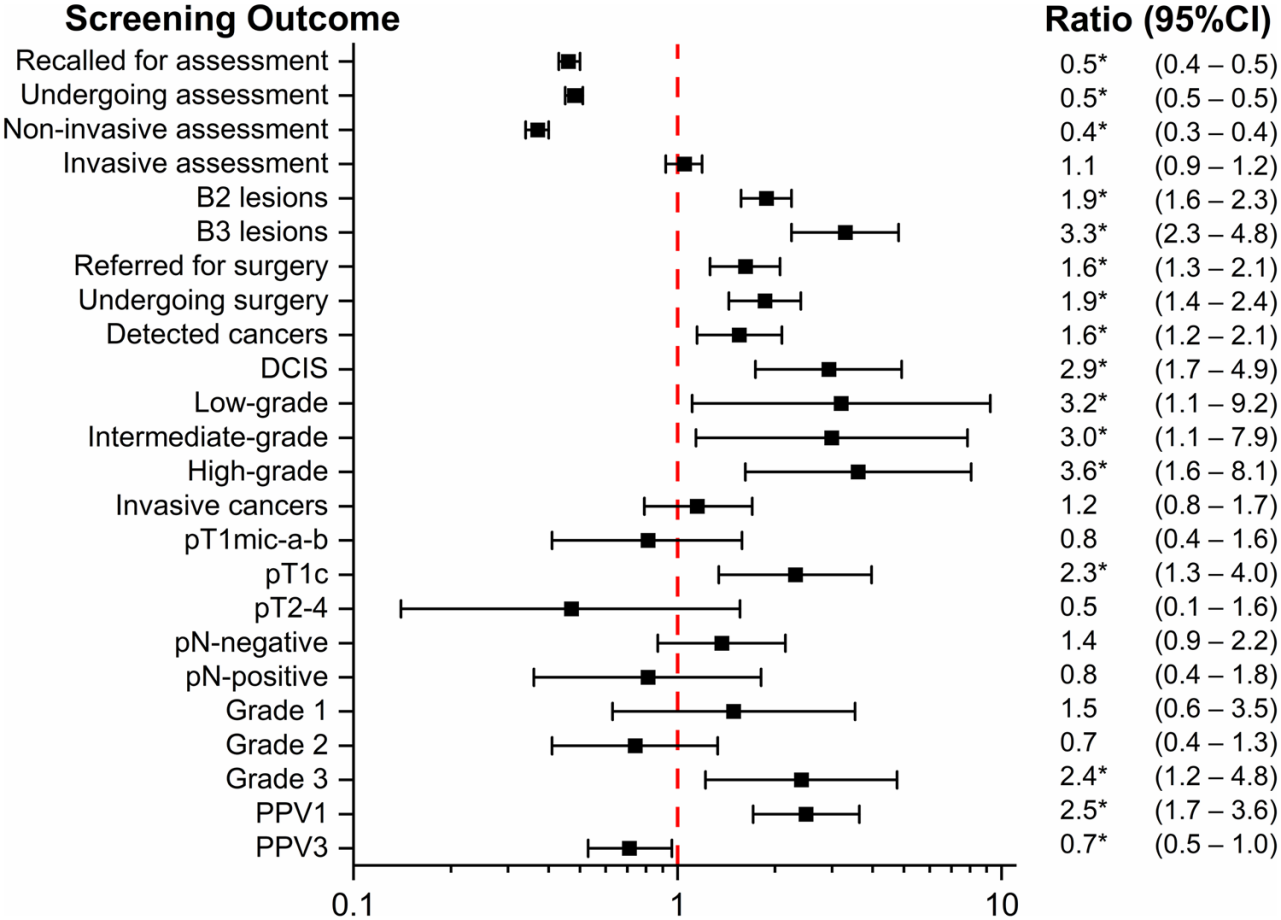
Screening outcome comparison between screening outcomes for women in the interventional and observational cohorts: forest plot of ratios. *Note*. B2 lesions, benign lesions from percutaneous biopsy; B3 lesions, lesions of uncertain malignant potential from percutaneous biopsy; DCIS, ductal carcinoma in situ; PPV1, positive predictive value of recall for additional imaging (non-invasive assessment); PPV3, positive predictive value of biopsy

Figure 5 shows the forest plot obtained from the ratios calculated by dividing each screening outcome rate/percentage in the overall interventional cohort by the corresponding value in the observational cohort.

With the use of DBT and, in high-density subgroups, supplemental US, the recall and assessment rates were more than 50% lower in the prospective interventional cohort, with the decrease limited to the non-invasive assessment rate. In contrast, the biopsy rate (invasive assessment) was more than 80% higher. In addition, the prospective interventional cohort showed a higher detection of B2 and B3 lesions and almost threefold higher yield of DCIS, with grades 2 and 3 being more frequent than grade 1.

**Fig. 6:**
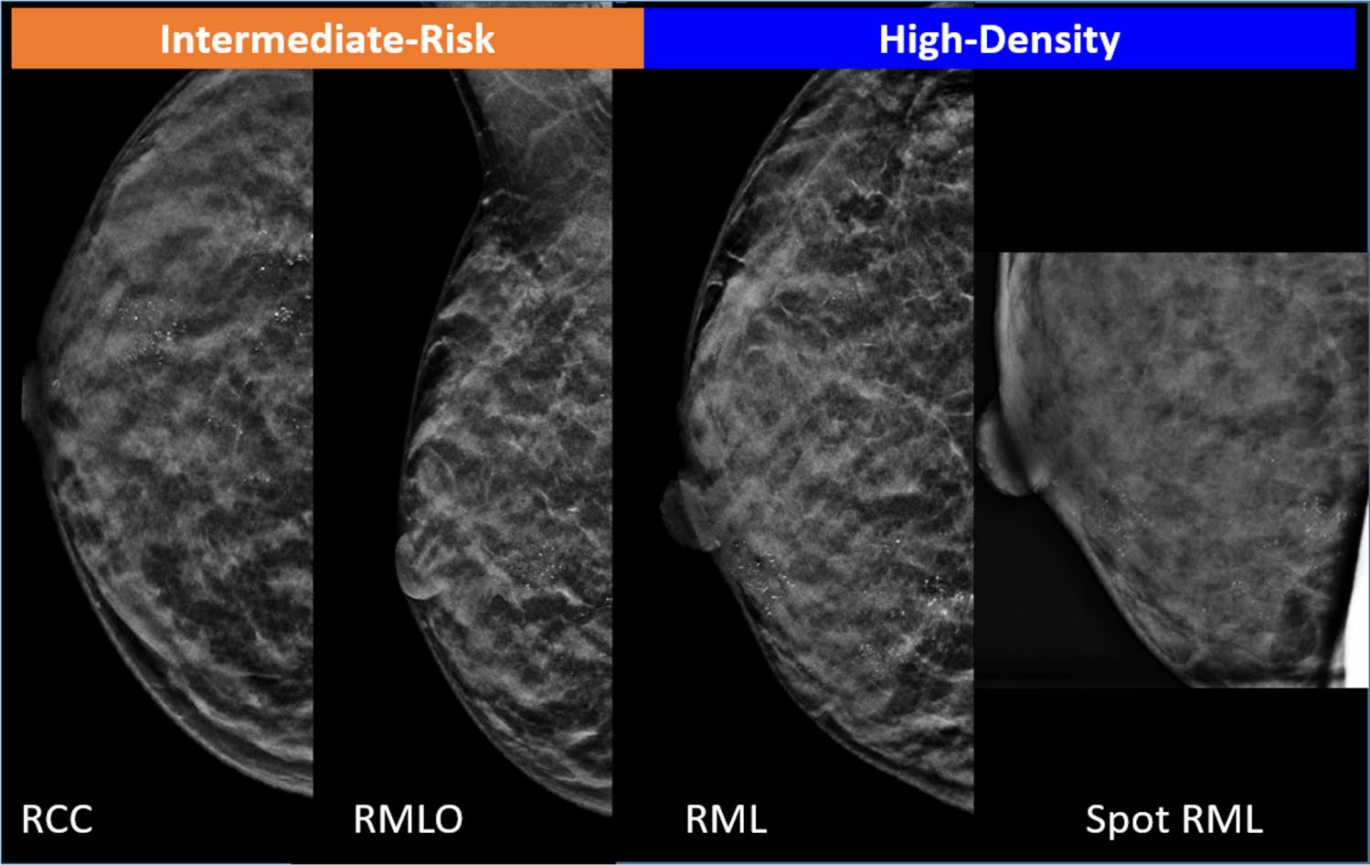
Example case of a grade 3 DCIS in the right breast of an intermediate-risk woman with high-density breasts: RCC, RMLO, RML contact views, and RML spot views. Synthetic images obtained from DBT. Pleomorphic sparse microcalcifications are present in the whole right breast. *Note*. DCIS, ductal carcinoma in-situ; RCC, right cranio-caudal; RMLO, right mediolateral-oblique; RML, right mediolateral; DBT, digital breast tomosynthesis

An example case of a grade 3 DCIS in the right breast of an intermediate-risk woman with high-density breasts is shown in Fig. 6.

Regarding invasive BC, the total detection rate was not significantly different from unity. However, there was a significantly higher detection of BC in the pT1c, with a ratio of 2.3, mainly due to grade 3 lesions. The positive predictive value of recall (PPV1) was 2.5 times higher, while the PPV of biopsy (PPV3) decreased by 30%.

## Discussion

The RIBBS study aims to evaluate the effectiveness of a personalized screening model based on individual risk and breast density, assessing its ability to reduce the cumulative incidence of advanced breast cancer as a surrogate for mortality reduction. The results from the prevalent screening provide promising indications for our ultimate goals.

The 25% VBD threshold used to distinguish between low- and high-density categories was defined based on preliminary analysis. Evidence from Larsen et al. [18], based on 508,536 screening examinations, indicates that the probability of interval cancers remains relatively stable between 15% and 25% VBD. The 25% VBD threshold set in the RIBBS study allowed for a balance between additional imaging needs and resource sustainability, with 18.5% of women requiring supplemental screening.

Women classified as low-risk had a lower prevalence of BC compared to those at intermediate-risk, who had a prevalence more than four times higher. Intermediate-risk women represent a significant portion of the cohort and are likely to play a crucial role in the effectiveness and sustainability of personalized screening models. Recruitment data are insufficient to assess the prevalence of invasive BC in high-risk women because of the small sample size. More complete data will be available after the rescreening period.

From the perspective of feasibility, several key findings related to screening intervals (annual or biennial) and additional screening tests (US or MRI) have emerged. First, low-risk women with low-density breasts constitute more than 50% of the interventional cohort and undergo biennial screening with DBT. This is a favorable outcome, as half of the women screened do not need intensified imaging and can use DBT as their only screening test, as recently suggested by the U.S. Preventive Services Task Force [7]. High-risk women who require combined rescreening with DBT and MRI constitute a small subgroup with a modest burden on radiology resources.

The initial effectiveness of the model is indicated by a more than doubled detection rate of pT1c BCs, along with the increased identification of grade 3 BCs. DBT has been shown to increase invasive cancer detection rates compared to mammography [41], but it carries an inherent risk of overdiagnosis, i.e. the detection of breast cancers that would not progress or become clinically significant [42]. A pT1c (size 1–2 cm) or grade 3 tumor cannot be considered overdiagnosis, given the young age of the women enrolled. These data support the expectation that rescreening will identify the fastest growing and clinically most significant tumors, reducing pT2-4 tumors [43]. This outcome is expected to occur in subsequent screens.

The increased detection of DCIS may increase the risk of overdiagnosis and overtreatment, but this finding is mitigated by the prevalence of grades 2 and 3. There is growing interest in nonsurgical management of DCIS, including active surveillance, to reduce overtreatment and provide patients with personalized management options. However, current biomarkers cannot accurately predict the risk of progression to invasive disease associated with calcification findings. However, high nuclear grade, comedo-like necrosis, and receptor status are key features of DCIS, influencing prognosis, risk of upstaging to invasive cancer at surgical excision, and eligibility for active surveillance [44].

The higher surgical intervention rate, almost doubled compared with DM screening, is related both to the higher cancer detection rate and an increase in B3 lesions due to the improved visibility of architectural distortions associated with DBT, a frequent semeiotic in this type of lesion.

The prospective interventional cohort also achieved a recall rate reduction greater than 50% compared with DM, especially for the ‘intermediate-risk, high-density’ subgroup. High recall rates are a significant issue in mammography screening in Italy and elsewhere. They can make the screening program less efficient and lead to subsequent non-adherence [45].

A quasi-experimental design is particularly suitable in those settings where random assignment to interventions is impractical, financially or logistically prohibitive, or socially and ethically unacceptable [34, 46]. This was the case for the RIBBS study. Notably, a comparable design has been successfully employed in prior studies, such as the Nijmegen study [47] and more recent cohort studies of mammography screening [48].

Quasi-experimental designs are pragmatic and allow for real-world evaluations with high external validity, especially in population-based settings. While their internal validity is lower than that of randomized controlled trials due to potential unmeasured confounders, in the RIBBS study this is partially mitigated by the similarity in baseline breast cancer (BC) incidence rates between the intervention and control cohorts. In addition, the cohorts were drawn from adjacent regions with similar socioeconomic profiles, which further supports comparability. The lack of risk and density data in the observational control cohort reflects the typical limitations of population-based screening programs. However, this limitation highlights the relevance and need for risk and density-based models such as RIBBS to effectively fill these gaps.

Another potential limitation of the study design is the time difference between the recruitment periods of the two cohorts. However, this does not appear to have introduced significant bias. Firstly, and contrary to expectations, the proportion of patients treated with neoadjuvant chemotherapy was low in both cohorts and not higher in the more recent interventional cohort (3.0% vs. 5.5%, data not shown), mitigating concerns about bias in tumor staging. Secondly, the modest decline in recruitment observed in the interventional cohort, which was entirely conducted during the COVID-19 pandemic, does not suggest significant selection bias. Finally, despite the observational cohort being recruited over a broader time frame, all examinations were performed with digital mammography, as the transition from film-screen mammography was completed in 2010–2011 [49]. This ensures that the comparison between screening strategies remains free from contamination bias.

In conclusion, although it is too early to draw definitive conclusions about the RIBBS study, the prevalence screening demonstrated that the use of DBT and, in high-density subgroups, supplemental US is both feasible and practical. The stratification criteria effectively identified subpopulations with different breast cancer prevalence, suggesting their potential utility. Importantly, while an increased detection rate of pT1c tumors alone is not sufficient to reduce the incidence of advanced breast cancer, it is a critical prerequisite to achieve this goal.

## Abbreviations

BC: Breast cancer
DBT: Digital breast tomosythesis
DM: Digital mammography
LR–LD: Low-risk low-density
LR–HD: Low-risk high-density
IR–LD: Intermediate-risk low-density
IR–HD: Intermediate-risk high-density
HR: High-risk
LTR: Lifetime risk
VBD: Volumetric breast density
